# Public health implications of computer-aided diagnosis and treatment technologies in breast cancer care

**DOI:** 10.3934/publichealth.2023057

**Published:** 2023-10-25

**Authors:** Kai Cheng, Jiangtao Wang, Jian Liu, Xiangsheng Zhang, Yuanyuan Shen, Hang Su

**Affiliations:** 1 Yantai Affiliated Hospital of Binzhou Medical University, Yantai, 264100, China; 2 Department of Electronics, Information and Bioengineering, Politecnico di Milano, Milan, Italy

**Keywords:** public health, breast cancer management, computer-aided diagnosis, machine learning, health care services, epidemiology, clinical medicine, multimodal technologies

## Abstract

Breast cancer remains a significant public health issue, being a leading cause of cancer-related mortality among women globally. Timely diagnosis and efficient treatment are crucial for enhancing patient outcomes, reducing healthcare burdens and advancing community health. This systematic review, following the PRISMA guidelines, aims to comprehensively synthesize the recent advancements in computer-aided diagnosis and treatment for breast cancer. The study covers the latest developments in image analysis and processing, machine learning and deep learning algorithms, multimodal fusion techniques and radiation therapy planning and simulation. The results of the review suggest that machine learning, augmented and virtual reality and data mining are the three major research hotspots in breast cancer management. Moreover, this paper discusses the challenges and opportunities for future research in this field. The conclusion highlights the importance of computer-aided techniques in the management of breast cancer and summarizes the key findings of the review.

## 1. Introduction

**Figure 1.**
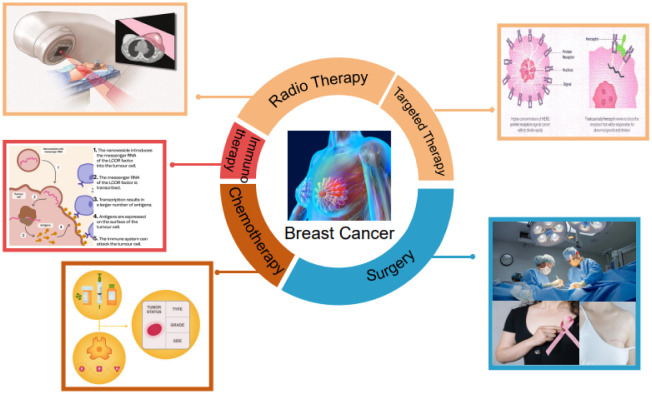
Overview of therapeutic modalities for breast cancer.

Breast cancer, a malignancy that originates from the mammary gland, is one of the root causes of cancer-related fatalities among female populations worldwide [1]. The etiology of breast cancer is multifactorial and encompasses a complex interplay of genetic, epigenetic and environmental factors [2]. The early detection and effective treatment of breast cancer is paramount for enhancing the disease prognosis. Regular breast cancer screening, such as mammography, as well as ultrasound and magnetic resonance imaging (MRI), can increase the likelihood of diagnosing breast cancer at an early stage [3], [4]. Moreover, prompt and appropriate treatment can impede the advancement of the disease and curtail the likelihood of metastasis. Effective treatment of breast cancer requires a multidisciplinary approach that encompasses a range of therapeutic modalities, including surgery, radiation therapy, chemotherapy, immunotherapy and targeted therapy, as shown in Figure 1 [5]–[7]. The choice of treatment depends on several factors, including the stage and classification of breast cancer, as well as the patient's overall health and personal preferences. In recent years, there has been an increasing trend towards the integration of computer-aided techniques in the field of breast cancer, with the purpose of improving the accuracy and efficiency of diagnosis and treatment [8], [9].

**Figure 2.**
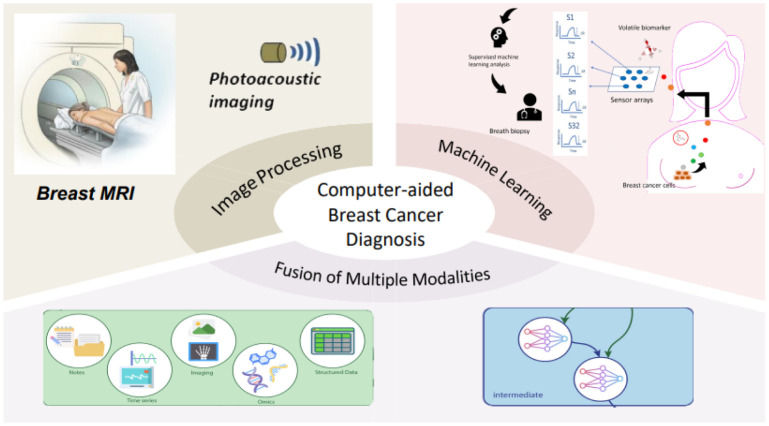
The computer technologies concerned with breast cancer diagnosis.

**Figure 3.**
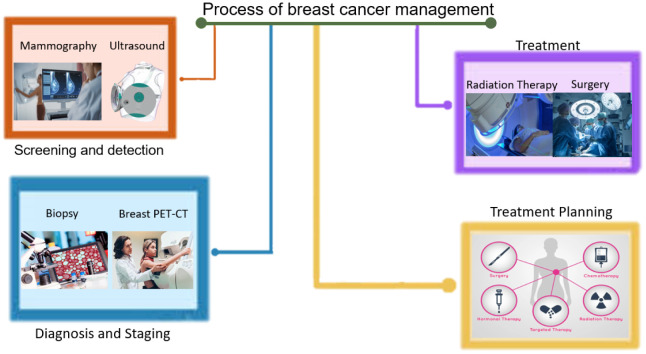
Diagram illustrating the integration of computer technology in the entire process of breast cancer management.

Computer technology has been integrated throughout the entire process of breast cancer diagnosis and treatment, as depicted in Figure 2. Computer-aided techniques have revolutionized the field of breast cancer management, offering an array of benefits that include improved diagnostic accuracy, treatment planning and simulation, and reduced treatment-related side effects [10]–[12]. These techniques leverage advanced image analysis and processing algorithms, as well as machine learning and deep learning algorithms, to provide a more sophisticated and accurate methodology in diagnosing and treating breast cancer [13]–[16]. As shown in Figure 3, computer-aided diagnosis involves the use of computer algorithms to analyze medical images, such as mammography, ultrasound, and magnetic resonance imaging (MRI), to enhance the accuracy and efficiency of breast cancer diagnosis [17]. Image analysis and processing techniques can be employed to identify the location, size and shape of lesions, while machine learning and deep learning algorithms can be drilled to recognize pathological features in images [18]. Multimodal fusion techniques that integrate different imaging modalities can also improve diagnostic performance [19]–[21]. Computer-aided treatment utilizes computer technology to optimize various aspects of breast cancer treatment. Radiation therapy planning and simulation can be performed using computer algorithms to determine the optimal radiation dose distribution to healthy tissues [22]. Computer-aided surgery guidance and navigation can aid in surgical planning and execution, as well as in the evaluation of surgical outcomes [23]–[25]. Computer-aided treatment monitoring and evaluation can be used to track the effectiveness of treatments and to predict patient outcomes. Precision medicine and molecular prognostics can be leveraged to evaluate disease progression and treatment efficacy, ultimately guiding the selection of the most appropriate treatment plan [26].

In recent years, several reviews have discussed the integration of technology in breast cancer diagnosis or treatment. In 2018, N I Yassin et al. [9] conducted a systematic review of the machine learning techniques for breast cancer computer-aided diagnosis, highlighting their application in images. Similarly, A Kajala and V Jain [27] also explored the application of machine learning in breast cancer diagnosis, demonstrating the efficiency and effectiveness of computer-aided technologies. Differently, focused on the role of artificial intelligence in the interpretation of breast cancer on MRI [28]. However, the latest examination concentrating on the broad-ranging implications of computer-aided diagnosis and treatment for breast cancer remains limited. This systematic review furnishes an all-encompassing overview of the present status of computer-aided techniques in breast cancer management, and serves as a valuable resource for researchers and healthcare professionals working in this field. Through a thorough literature search and evaluation according to the Preferred Reporting Items for Systematic Reviews and Meta-Analyses (PRISMA) guidelines, this paper offers insights into recent advancements in computer-aided diagnosis and treatment for breast cancer. It highlights the various techniques that are being used, including image analysis and processing, machine learning algorithms, multimodal fusion, and radiation therapy planning and simulation, to improve diagnostic accuracy and optimize treatment planning and simulation, as well as to reduce treatment-related side effects. Furthermore, this review discusses the challenges and future directions of computer-aided diagnosis and treatment in breast cancer management, offering insights into potential avenues for further research and development in the field.

The subsequent sections of this systematic review article are organized as follows: In Section 2, a thorough description of the methodology utilized in conducting the review is provided. The recent advancements in computer-aided diagnosis in breast cancer management are comprehensively reviewed in Section 3. Section 4 explores the advancements in computer-aided treatment for breast cancer management. The benefits and future directions of computer-aided techniques in breast cancer management are analyzed in Section 5. The review is concluded in Section 6, where a summary of the key outcomes is provided, and recommendations for future research are given.

## 2. Materials and methods

### 2.1. Search strategy

In line with the Preferred Reporting Items for Systematic Reviews and Meta-Analyses (PRISMA) guidelines, this systematic review was carried out to assess recent advancements in the realm of computer-aided diagnosis and treatment in breast cancer management. A comprehensive search of six databases, including Cochrane Library, Scopus, MEDLINE, Web of Science, PubMed and EMBASE, was conducted to identify relevant studies published between January 15, 2018 and January 15, 2023. The search was limited to articles and was guided by a well-defined search strategy that utilized appropriate MeSH terms and keywords, such as “computer,” “breast cancer,” “diagnosis,” and “treatment.” This approach was implemented to ensure the validity and reliability of the studies involved in the review.

### 2.2. Study selection

The eligibility criteria employed in this systematic review included the following: (1) original research articles, (2) studies written in the English language and (3) studies assessing the application of computer technology in breast cancer management. Studies failing to meet these criteria were excluded, including: (1) studies with non-relevant objectives, (2) case reports and (3) studies that described advancements without examining their impact on breast cancer.

### 2.3. Risk-of-bias assessment

The quality of the incorporated studies was assessed through a systematic evaluation of their risk of bias. The Cochrane Risk of Bias tool was utilized for randomized controlled trials, while the Newcastle-Ottawa Scale was employed for observational studies. The quality was carried out independently by two reviewers, with any discrepancies addressed through consultation with a third reviewer to ensure consistency and reliability of the results.

### 2.4. Heterogeneity and sensitivity analysis

**Figure 4.**
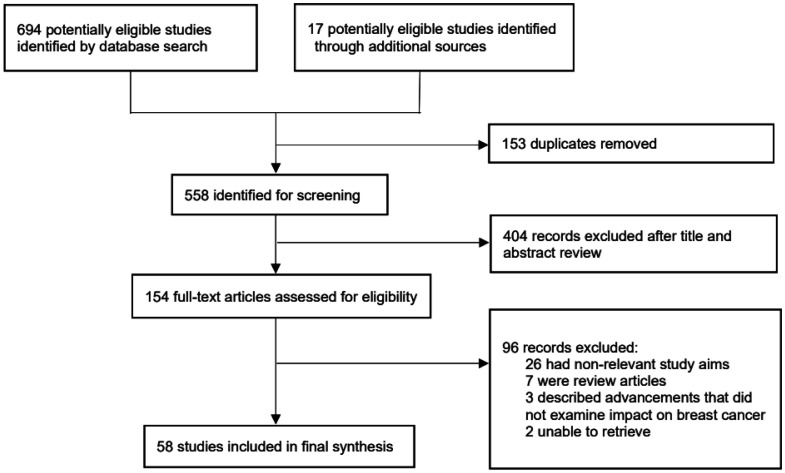
PRISMA flowchart of the search process.

Heterogeneity among the selected studies was quantified using the I² statistic. Studies with I² values above 50% were considered to have substantial heterogeneity. Sensitivity analysis was conducted to assess the robustness of the review findings. This involved excluding one study at a time and assessing its impact on the overall results. This step ensured that no single study disproportionately influenced the overall conclusions of the review. In total, 694 records were identified from searches in all databases, and 17 additional records were identified through additional sources; a total of 58 entries were incorporated into the study. The PRISMA flowchart of the search process is presented in Figure 4.

## 3. Advancements in computer-aided diagnosis.

The recent advancements in computer-aided diagnosis for breast cancer management have been significant. The use of computer technology has revolutionized the way breast cancer is diagnosed, improving the accuracy and efficiency of the diagnostic process. Through image analysis and processing, machine learning algorithms and multimodal fusion, computer-aided techniques have made it possible to detect breast cancer at earlier stages, leading to improved patient outcomes and reduced healthcare costs. In particular, datasets play a crucial role in the development and optimization of AI models for breast cancer diagnosis. These datasets contain a vast amount of breast cancer image data and corresponding diagnostic labels, which can be used to train and optimize AI models. Table 1 presents representative studies that used datasets for breast cancer diagnosis. These advancements in computer-aided diagnosis have paved the way for a more precise and effective approach to breast cancer management.

**Table 1.** Overview of representative studies that used datasets for breast cancer diagnosis.

**Authors**	**Year**	**Dataset**	**Methods**	**Performance results (%)**
S Charan et al. [29]	2018	Mammograms-MIAS dataset	CNN	ACC = 65
L Tsochatzidis et al. [30]	2019	DDSM-400; CBIS-DDSM	CNNs	AUC(1) = 83, ACC(1) = 74, AUC(2) = 78, ACC(2) = 74
L Shen et al. [31]	2019	CBIS-DDSM	CNN	AUC = 88
W M Salama and M H Aly [32]	2021	BreaKHis databas	End-to-end fully CNNs	ACC = 99.43, AUC = 99.22, Sen = 99.12, Pre = 98.99
Ü Budak et al. [33]	2019	BreaKHis dataset	CNN and Bi-LSTM	ACC = 91.90
H Aljuaid et al. [34]	2022	BrakeHis dataset	DNNs and transfer learning	ACC = 97.81

### 3.1. Image analysis and processing techniques

Image processing techniques can analyze mammogram, ultrasound images and magnetic resonance imaging, helping radiologists determine the location, size and shape of lesions.

Mammogram is the most commonly used imaging technique for detecting breast cancer, and the hybrid feature selection approach proposed in the study uses image processing techniques to enhance its accuracy in diagnosing breast tumors. A proposed hybrid feature selection approach combines a support vector machine recursive feature elimination with correlation bias reduction algorithm and a similarity-based learning algorithm called Q for benign-malignant classification. The system's performance, as demonstrated by its accuracy (98.16%), sensitivity (98.63%), specificity (97.80%) and computational time (2.2 s), surpasses that of existing computer-aided diagnosis systems [35]. M Mehmood et al. [36] improved the accuracy of breast tumor diagnosis by utilizing image processing techniques. The mammograms were preprocessed employing contrast-limited adaptive histogram equalization and isolated through threshold detection and morphological operations. The extracted texture, shape and gray-level co-occurrence matrix characteristics were classified by support vector machine (SVM) while adaptive neuro fuzzy inference system (ANFIS) was deployed for differentiation between ductal carcinoma in situ (DCIS) and lobular carcinoma in situ (LCIS). The results showed high accuracy of 98.95% and 98.01% for standard and nonstandard mammograms, respectively, through cubic support vector machines (CSVM) and promising results in terms of mean square error (MSE) of 0.01866, 0.18397, and 0.19640 for DCIS and LCIS differentiation during the phases of training, examination and validation through ANFIS. However, there are still detection metrics that proved less useful. For example, an evolutionary approach was developed for identifying and categorizing breast cancer utilizing machine learning and image processing [37]. AlexNet was employed to extract features from the data with an accuracy of 89.00%.

**Figure 5.**
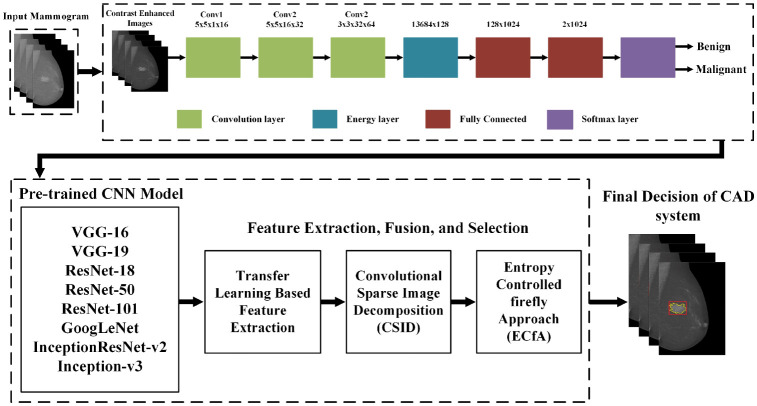
Framework of the proposed approach for breast cancer detection and classification [40].

The use of AI-assisted computational techniques and image processing in mammography images leads to improved accuracy and efficiency in breast cancer diagnosis [38]. Q Liu et al. [39] proposed a robust image segmentation method that accounts for an accuracy of 92.9% and has potential in independent variables through interval analysis and the use of the Laplacian of Gaussian filter. S Maqsood et al. [40] presented a deep learning system that leverages a modified contrast enhancement method and alienable texture convolutional neural network to ascertain breast cancer in mammogram images, achieving an average accuracy of 97.49%, and its framework is as shown in Figure 5. These results indicate the potential of deep learning algorithms to improve mammography screening tools and diminish the incidence of false positive and false negative outcomes. The use of computational techniques and image processing with AI was emphasized as crucial to improving diagnosis accuracy and efficiency in [41]. This study also highlights mammography as the primary exam for early detection and the beneficial impact of technology advancements in the field.

In terms of other computer-aided image analysis technology, Pavithra et al. developed a CAD system for the detection and categorization of breast cancer utilizing breast ultrasound (BUS) imaging [42]. The system comprised four stages: pre-processing, segmentation, feature extraction and classification. The pre-processing step utilized SRAD to eliminate speckle noise and employed active contour-based segmentation to identify the ROI. The extracted texture features were then classified into Normal, Benign or Malignant categories using K-nearest neighbors (KNN), decision tree or random forest classifiers, and their accuracy was compared. Additionally, a computational thermal model of breast cancer was developed grounded on high-resolution infrared images, 3D breast surface geometries and internal tumor definition obtained from a breast cancer patient [43]. The model was calibrated to the patient's clinical data and evaluated the thermal peculiarities of the patient's triple negative breast cancer with high metabolic heat generation rates. The experimental results were precise to the patient's distinctive molecular subtype of breast cancer, lesion size and stage, thus rendering their potential applicability to analogous aggressive instances.

### 3.2. Machine learning and deep learning algorithms

Image analysis and processing involves the examination of images to extract meaningful information and insights from them. Machine learning algorithms, including deep learning algorithms, can automate the image analysis process by training algorithms to recognize patterns and make predictions based on input data.

The possibility of employing deep learning methodologies, notably convolutional neural networks (CNNs), for detecting breast cancer in mammogram images was underscored in [29], [44]. The study used the mammograms-MIAS dataset and showed promising results in classifying normal and abnormal breast images. Further optimization of CNN architectures is expected to enhance the accuracy of breast cancer detection, with proper segmentation being crucial for efficient feature extraction and classification. An innovative automated computer-aided diagnosis system was presented for breast cancer diagnosis, characterized by high precision and minimal computational demands. The use of deep convolutional neural networks in computer-aided diagnosis of breast cancer was also examined in [30], evaluating their performance on two mammographic datasets through fine-tuning pre-trained networks and training from scratch.

**Figure 6.**
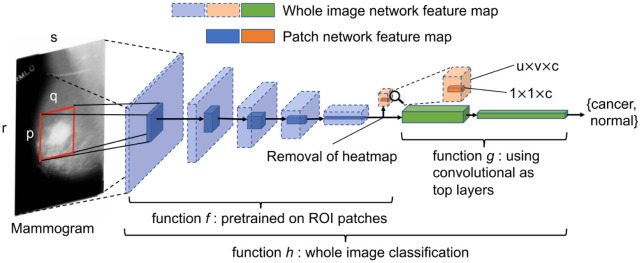
Converting a patch classifier to an end-to-end trainable whole image classifier using an all convolutional design [31].

Several studies have aimed to increase the accuracy of breast cancer detection utilizing deep learning algorithms and image processing techniques in mammography images. In [31], an “end-to-end” deep learning algorithm was developed and demonstrated superior performance compared to previous methods, with per-image AUC scores of 0.88 on the Digital Database for Screening Mammography (DDSM) and 0.98 on the INbreast database (see Figure 6). This study highlights the potential of deep learning methods in enhancing clinical tools and reducing false positive and false negative results in mammography-based breast cancer detection. The research of S Chaudhury et al. [45] proposed a framework for breast cancer detection in mammography images, incorporating the contrast limited adaptive histogram equalization (CLAHE) approach and categorization through fuzzy SVM, Bayesian classifier, and random forest. In [32], a novel framework that combined various segmentation and classification models, including MobileNetV2, InceptionV3, DenseNet121, VGG16 and ResNet50, was introduced, achieving top results on the DDSM dataset with 98.87% accuracy and a computational time of 1.2134 s.

**Figure 7.**
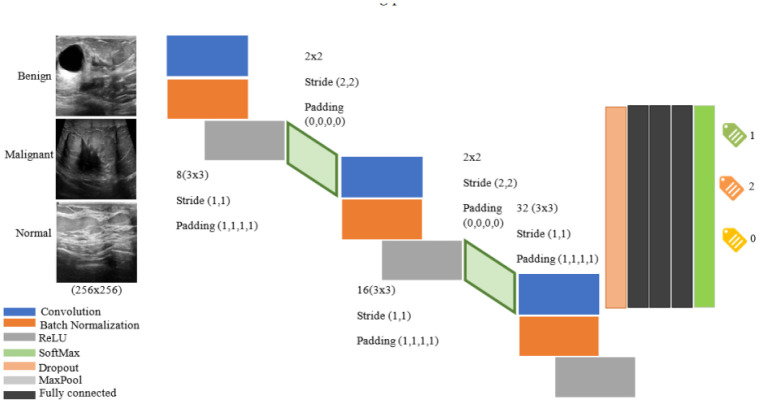
The architecture provided by CNN for classifying or diagnosing tumor type and disease[47].

Studies have been conducted with the aim of formulating computer-assisted models for diagnosing breast cancer utilizing ultrasound images. In [46], a convolutional neural network (CNN) was utilized to develop a computer-assisted model for breast cancer diagnosis using ultrasound images. The study analyzed 5000 images and found that the highest-performing model was InceptionV3, with an AUC of 0.905. This model outperformed the diagnostic accuracy of sonographers, demonstrating statistically significant improvement with an AUC of 0.913, indicating the high accuracy of the CNN-based prediction model in breast cancer diagnosis. In [47], a machine learning method was developed for early detection and diagnosis of breast cancer using ultrasound images, whose architecture is as shown in Figure 7. The study employed various classification techniques, including K-nearest neighbor, support vector machine, decision tree and Naive Bayes, as well as a convolutional neural network (CNN) for direct classification of breast cancer based on ultrasound images. The outcomes showed an accuracy of 99.8% for the training set and 88.5% sensitivity in diagnosis validation.

In 2022, S Bourouis et al. [48] developed a computer-assisted diagnosis system that utilizes a combination of the grey wolf optimization (GWO) algorithm and a wavelet neural network (WNN) for detecting abnormalities in breast ultrasound images. The system involves preprocessing of the images, extraction of morphological and texture features and classification using the GWO-tuned WNN, achieving an accuracy of 98%. A mobile phone-based system was also presented by X Qi et al. [49] to enhance the precision of breast cancer diagnosis through ultrasonography images. This system, consisting of three subsystems for reducing noise, classifying images and detecting anomalies, was trained and evaluated on over 18,000 images and 2,400 ultrasound reports. The utilization of deep convolutional neural networks and generative adversarial networks resulted in performance comparable to that of human experts.

Efforts have been made to improve the accuracy of detecting and diagnosing breast lesions in magnetic resonance imaging (MRI) and dynamic contrast-enhanced MRI (DCE-MRI) using machine learning systems. In [50], a machine learning system was evaluated for its ability to discriminate between malignant and benign breast lesions using MRI data from a single institution. The system merged radiomic features with a support vector machine to produce a lesion signature and achieved an AUC value of 0.89 and 99.5% sensitivity, with 9.6% fewer suggested biopsies compared to actual clinical decisions. A Tahmassebi et al. [51] assessed the use of machine learning with multiparametric MRI (mpMRI) to predict pathological complete response (pCR) and survival outcomes in breast cancer patients receiving neoadjuvant chemotherapy (NAC). The results indicated that machine learning with mpMRI is a stable and accurate predictor of pCR and survival outcomes, with the XGBoost classifier achieving the highest accuracy. In [52], an AI system was developed and evaluated for detecting and diagnosing lesions in DCE breast MRI. The system, trained using RetinaNet, showed improved diagnostic performance compared to radiologists with a sensitivity of 0.926, specificity of 0.828 and an AUC of 0.925. The system also enhanced the diagnostic performance of human readers, leading to a significant increase in AUC when used as a tool. Ü Budak et al. [33] put forth a new end-to-end model on the basis of bidirectional long short-term memory (Bi-LSTM) and fully convolutional network (FCN) for the early diagnosis of breast cancer. The model extracted high-level features from high-resolution images and its accuracy was evaluated using the publicly available BreaKHis database, with the results demonstrating improved accuracy compared to previous methods. Later, J Zheng et al. [53] introduced a novel DLA-EABA algorithm that combined deep learning and advanced computational techniques to detect breast cancer. By integrating machine learning approaches with feature selection and extraction techniques, the study achieved high accuracy in breast cancer diagnosis using various imaging modalities, including mammography, ultrasound, MRI and digital breast tomosynthesis. The deep learning framework, which consisted of convolutional layers LSTM, max-pooling layers, and a completely connected layer with a softmax layer, achieved promising results with an accuracy of 97.2%, sensitivity of 98.3%, and specificity of 96.5%. More recently, H Aljuaid et al. [34] presented a computer-aided diagnosis method that combined deep neural networks and transfer learning on a public dataset to classify breast cancer. The method achieved high accuracy results in both binary (malignant/benign) and multi-class classification, with an average accuracy of 99.7% for binary classification and 97.81% for multi-class classification using ResNet.

### 3.3. Fusion of multiple modalities

The accuracy of classification for pathology images has been profoundly enhanced by means of deep learning algorithms, however, relying solely on a single modality of pathology images still falls short of meeting the needs of clinical practices in terms of accuracy for breast cancer classification. Therefore, combining information from multiple imaging modalities is crucial in the diagnosis of breast cancer.

Researchers have made progress in raising the accuracy of breast cancer diagnosis through multimodal ultrasound. By combining various types of ultrasound imaging and machine learning techniques, high diagnostic performance comparable to human observers has been achieved, offering a promising approach for the future. L R Sultan et al. [54] investigated the combination of grayscale and Doppler ultrasound images to differentiate between benign and malignant breast lesions, resulting in an AUC of 0.96, sensitivity of 92% and specificity of 95% after pruning weakly learned cases. M A Mohammed et al. [55] suggested a method for automating the characterization of breast cancer in ultrasound images using multi-fractal dimensions and backpropagation neural networks, achieving a precision rate of 82.04%, sensitivity of 79.39% and specificity of 84.75%. R Huang et al. [56] come up with a novel framework, AW3M, characterized by four types of sonography in a multi-stream CNN model, incorporating self-supervised consistency loss and optimal weight learning via reinforcement learning approaches, as well as a recovery block intended to address absent modalities during testing. The results showed that AW3M outperforms existing methods and can handle missing data, making it a promising approach for breast cancer diagnosis.

**Figure 8.**
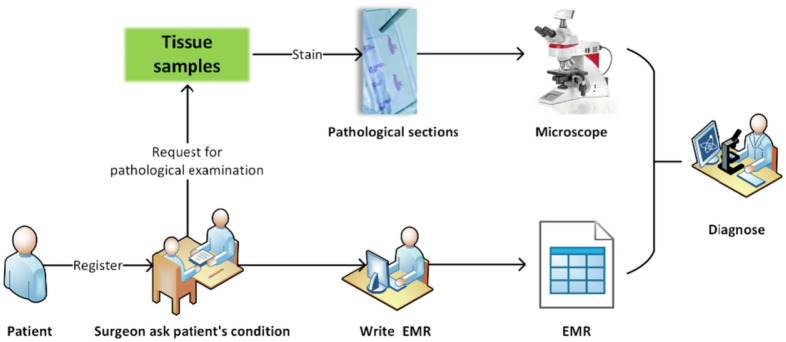
A simple brief introduction to pathological diagnosis workflow in the hospital [57].

The accuracy of breast cancer diagnosis was improved through the implementation of a multimodal fusion-based computer-aided diagnosis system that incorporated MRI and mammography. R Mokni et al. in [20] proposed a computer-aided diagnosis system that fused information from dynamic contrast enhanced magnetic resonance imaging (DCE-MRI) and digital mammographic images. The system utilized the GLIP local feature descriptor and canonical correlation analysis (CCA) to emphasize the relationship between the two modalities, resulting in high diagnostic performance with an AUC of 99.10% using the radial basis function neural network classifier. R Yan et al. in [57] proposed a richer fusion network for the classification of benign and malignant breast cancer based on multimodal data, including pathological images and structured data from clinical electronic medical records (Figure 8). The method used a denoising autoencoder to boost the dimensionality of structured data and extract a multi-tiered feature representation of the pathological image. The proposed method outperformed previous methods with an average classification accuracy of 92.9% and has potential for practical use in clinical breast cancer diagnosis.

## 4. Advancements in computer-aided treatment

After a comprehensive and systematic diagnosis, an effective and personalized treatment plan needs to be developed. The recent advancements in computer-aided treatment for breast cancer management encompass a range of techniques, including planning and simulation in radiation therapy, surgical guidance and navigation, monitoring and assessment of treatment response and integration with precision medicine. These techniques represent a significant step forward in the management of breast cancer, enabling more effective and personalized treatment options. In computer-aided breast cancer treatment, datasets play a crucial role in facilitating medical professionals to make more informed decisions. These datasets typically consist of large collections of breast cancer-related data. Table 2 highlights the different types of data that were utilized to assist breast cancer treatment.

**Table 2.** Overview of representative studies that used datasets for breast cancer treatment.

**Authors**	**Year**	**Dataset**	**Methods**	**Performance results (%)**
H Duanmu et al. [58]	2020	ISPY1 Clinical Trial dataset	CNN networks	AUC = 80 Sen = 68, SP = 88
M Byra et al. [59]	2020	ImageNet dataset	Transfer learning	AUC = 79.7
L L De Boer et al. [60]	2018	Real-time DRS data	Support vector machine	ACC=93, MCC=87
S A Kulkarni et al. [61]	2021	3D volumetric image dataset	ROC analysis	AUC(1) = 91 AUC(2) = 90, AUC(3) = 94
V Chaurasia et al. [62]	2018	Wisconsin Breast Cancer datasets	RBF Network & Cox regression Kaplan–Meier survival analysis	ACC = 96.77
Á Ösz et al. [63]	2021	proteome-level breast cancer dataset		AUC = 74

### 4.1. Planning and simulation in radiotherapy and chemotherapy

Significant advancements have been made in the field of computational radiology and radiology simulation for the purpose of planning and simulating radiotherapy and chemotherapy for breast cancer patients. These advancements include the ability to predict patient responses to novel adjuvant therapies, ultimately leading to enhanced treatment outcomes.

The development of computer technology has enabled the efficient analysis of large and complex datasets through the use of bivariate and multivariable regression calculations and modeling. In a study by J C Hong et al. [64], the authors aimed to evaluate the mean heart dose (MHD) of adjuvant radiation therapy (RT) for breast cancer and the approximated risk of RT-associated cardiotoxicity in female populations. The study found that MHD varied based on the RT technique and was affected by patient positioning and breathing during RT. The total risk of cardiotoxicity was moderate, with 3.5 excess events per 1000 patients, and varied based on the RT technique employed. In another study by O Sager et al. [65], the effectiveness of adaptive radiotherapy (ART) was assessed through rescheduling the tumor bed boost using repetitive CT simulations after whole breast irradiation (WBI) for patients with seroma. The study included 48 patients, and two RT therapeutic regimes were formulated for each patient to track changes in seroma and boost target volume. The results proved a significant reduction in seroma volume and critical organ doses with ART, suggesting the benefits of ART in reducing seroma and critical organ doses for patients undergoing WBI. These findings have important implications for the optimization of RT treatment planning and the improvement of patient outcomes.

In radiotherapy, the high doses of radiation can potentially cause damage to the heart and blood vessels, making it crucial to carefully monitor patients receiving radiotherapy for breast cancer and take measures to minimize the risk of cardiac toxicity. In this regard, J W Jung et al. [66] developed a novel automated methodology for segmenting the cardiac substructures in radiotherapy CT images. The findings of the study indicate that the variance in doses for simulated breast radiotherapy between automatic and manual contours was minimal. The use of more than ten atlases did not significantly improve performance, and manual guide points did not significantly enhance the method's efficacy. In radiation oncology, the current standard treatment approach involves prescribing protocols based on the strength of general results of clinical tests, which lacks individuation and fails to account for patients' individual responses. The integration of mathematical models into radiation oncology has the potential to improve treatment evaluation and lead to enhanced patient outcomes through individualized adaptive radiation therapy (RT) [67]. By using mathematical models to simulate a patient's tumor growth and forecast treatment response, dynamic biomarkers can be developed for RT, enabling individualized treatment for patients.

**Figure 9.**
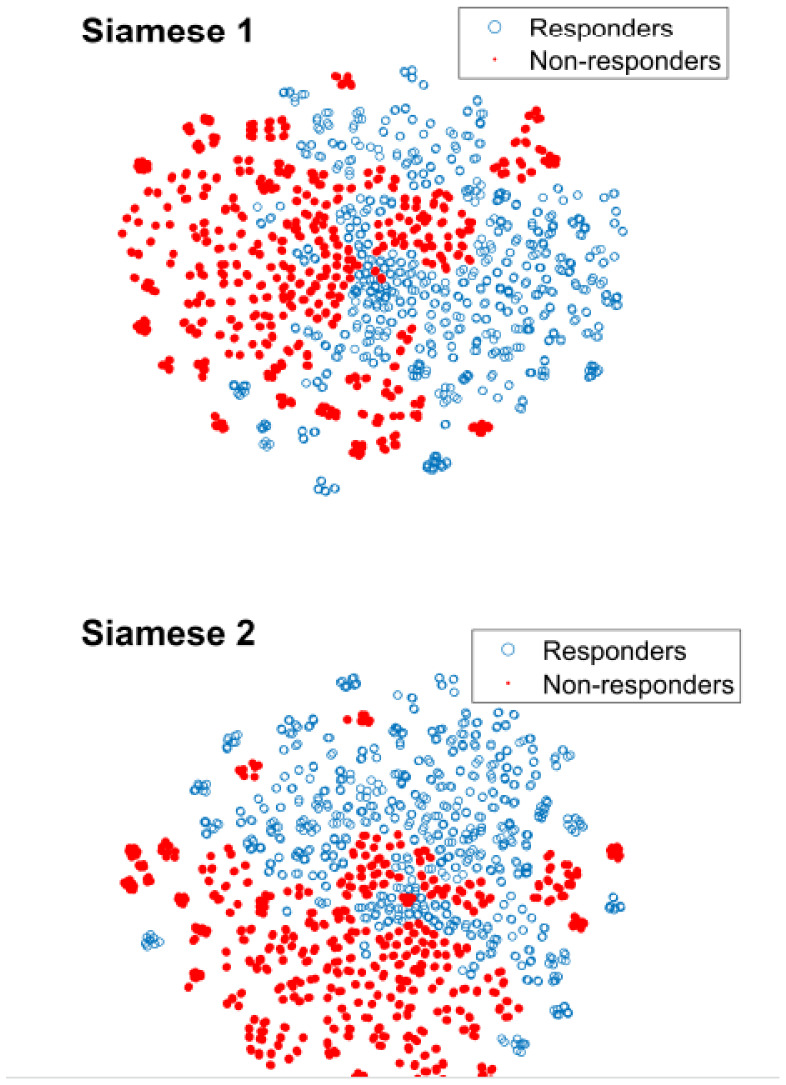
Visualizations of feature distribution with the t-SNE algorithm. The Siamese models were trained using features extracted from the Inception-ResNet-V2 (no fine-tuning) based on US image pairs. Each pair contained images of the same tumor collected before and after the neoadjuvant chemotherapy [68].

Machine learning algorithms combining imaging data molecular data, and demographic data have been used to predict breast cancer patients' response to neoadjuvant chemotherapy. H Duanmu et al. [58] employed a convolutional neural network with a novel approach that combined 3D MRI imaging data, molecular data and demographic data to forecast the probability of pathological complete response to neoadjuvant chemotherapy in breast cancer patients, achieving high accuracy and AUC values that outperformed models using imaging data only or conventional concatenation models. M Byra et al. [59] suggested a promising deep learning approach using ultrasound imaging, utilizing transfer learning with convolutional neural networks and comparing the results with a traditional method established on handwrought morphological features. The study results showed promising performance, with the best model achieving an AUC of 0.847 in the comparison of ultrasound images before and after treatment. In addition, L Yang et al. [68] developed a prediction model using a combination of gene expression and a machine learning algorithm, which showed significant differences in pCR rates between sensitive and insensitive groups, with the Naive Bayes algorithm being found to have the highest predictive value. The model had a sensitivity of 84.5 and specificity of 62%, and the 2D feature visualizations for training are as illustrated in Figure 9.

The field of surgical guidance and navigation for breast cancer has witnessed significant advancements through the integration of computer technology, including the utilization of augmented reality, virtual reality and image-guided techniques. These innovations have facilitated more precise and efficient surgical procedures, ultimately resulting in improved patient outcomes.

**Figure 10.**
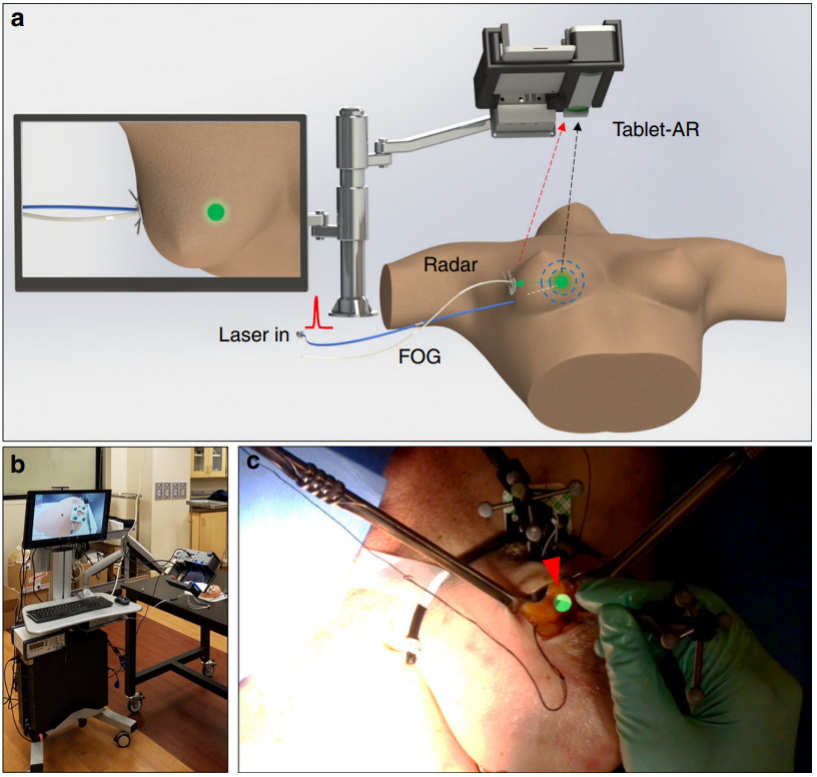
Using a fiber optoacoustic guide and an augmented reality (AR) system to locate the tumor and guide for fast and precise tumor removal [69].

**Figure 11.**
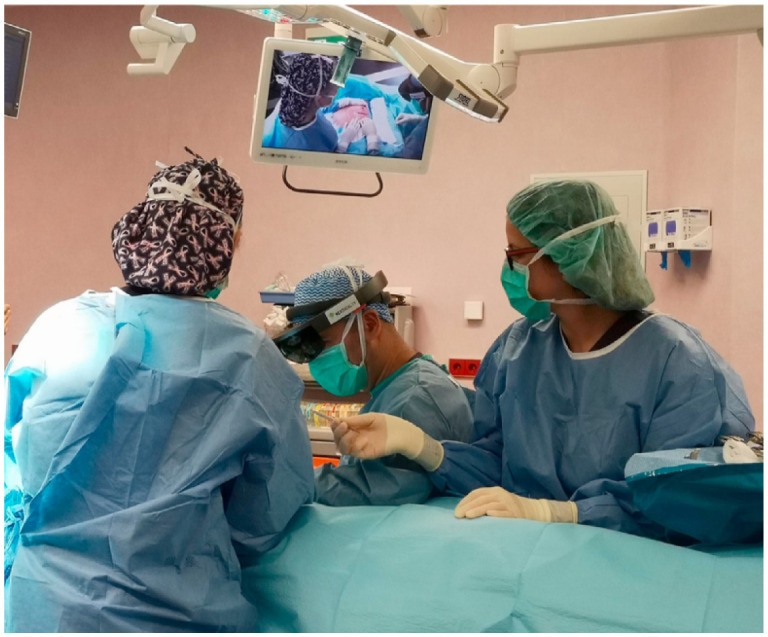
In action: surgeon wearing Hololens headset at the surgical theater [70].

**Figure 12.**
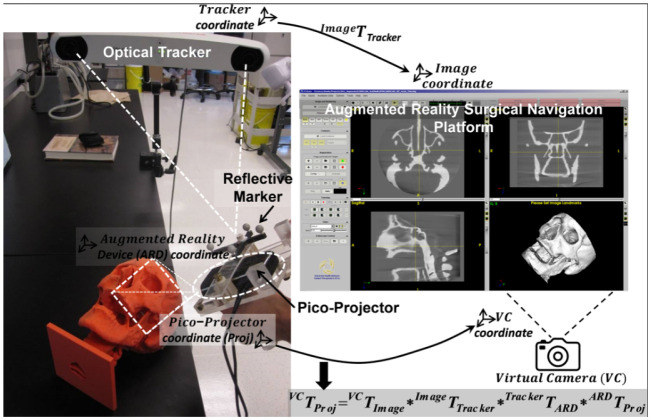
Prototype augment reality surgical navigation platform consists of optical tracking system and tracked pico-projector [72].

Augmented reality (AR) technology has become increasingly popular in breast cancer surgeries, providing precise and efficient outcomes as demonstrated in several studies. L Lan et al. [69] developed a fiber optoacoustic guide (FOG) with AR for precise and efficient breast cancer surgery, as illustrated in Figure 10. The FOG was implanted in the tumor and transmitted acoustic waves, which were captured by ultrasound sensors to provide real-time visual feedback to the surgeon via AR, allowing for accurate and quick tumor removal with minimal interference. The successful implementation of this technology in a cadaver study demonstrated the potential for reduced re-operation rates and shorter surgery times. Advancements in 3D spatial technology and AR, powered by high-tech computer science, have rapidly progressed breast cancer imaging and led to the creation of less invasive medical procedures. P F Gouveia et al. [70] presented the first use of digital, non-invasive AR for breast cancer surgery, with a 57-year-old woman undergoing breast surgery using AR for localization, as presented in Figure 11. The method was compared with traditional carbon tattooing, with the surgeon using a Hololens headset for visualization. The experiment showed a successful overlap of previous marks and visualization of the tumor. B Allison et al. [71] presented Breast3D, a mammographic image analysis system that utilizes extended reality (XR) technology to reconstruct CT and MRI scan data for breast cancer diagnosis and surgical planning. Breast3D provides a promising solution for XR within diagnostics of 3D mammographic modalities, which has been underutilized in the past. Moreover, H H Chan et al. [72] developed a new AR surgical positioning system that enhances visualization of concealed anatomy during surgery by projecting virtual images onto the operative site, demonstrating the clinical adaptability and pinpoint precision of the AR surgical navigation system with accuracy tested to be <1mm using a phantom (Figure 12).

### 4.2. Surgical guidance and navigation

Virtual reality (VR) technology has been applied in various aspects of breast cancer surgery, including non-invasive localization of occult breast cancer, reducing psychological distress in patients during chemotherapy, and improving pain, range of motion, muscle strength, functionality and nervousness of movement in post-surgery patients. A retrospective study evaluated a novel virtual localization technique for occult breast cancer, which is a proof-of-concept for a non-invasive tool [73]. The method involved preoperative MRI and intraoperative 3D optical scanning, and was compared with traditional radioisotopic localization. Results showed successful tumor localization with a mean cutaneous distance of 1.4 cm in patients with low breast volume and 2.8 cm in those with large breast volume. Similarly, a study in Italy compared the effects of VR and music therapy (MT) on reducing psychological distress in breast cancer patients during chemotherapy [74]. Both VR and MT were found to be effective in reducing anxiety and improving mood, with VR being more effective in alleviating anxiety, depression and fatigue compared to MT. The objective of the study by Ö Feyzioğlu et al. [75] was to compare the impact of Kinect-based VR therapy and standardized physiotherapy on various parameters, including pain, sphere of motion, muscle strength, functionality and nervousness of movement in women who underwent breast cancer surgery. Results showed significant improvements in all areas for both groups, with the standardized physiotherapy group showing more improvement in functionality and the Kinect-based VR therapy group displaying greater improvement in fear of movement.

Doctors can use computer-generated 3D image models to evaluate the position, size and shape of breast cancer before surgery, which provides guidance for the planning and execution of the surgery. An innovative approach using multimodality 3D whole-tumor imaging data has been developed to analyze angiogenic heterogeneity in breast tumor xenografts [76]. Computer-generated 3D image models were used to visualize the spatial heterogeneity of whole-tumor hemodynamics and intravascular oxygenation, which provided guidance for surgeries. The approach contributed to the understanding of the abnormal organization and hemodynamics of the tumor microvasculature, thus enhancing inter- and intra-tumor heterogeneity. This hybrid image-based modeling framework served as a foundation for a “cancer atlas” that could be applied to the study of other tissues and diseases. Research was also conducted to assess the efficacy of Micro-CT in analyzing breast cancer specimens [77]. The researchers found that Micro-CT images closely matched the size and shape of cancers seen at dissection and revealed additional information on cancer location not seen in traditional pathology methods. Additionally, Micro-CT was able to identify margin-positive cancers with higher accuracy and provided full 3D images of the specimens in minutes. In [78], patchy polymeric photoacoustic contrast agents and photoacoustic computed tomography were integrated to develop a non-invasive method for detecting intratumor heterogeneity in breast cancer. The technology utilized specific agents to distinguish between different receptor types in tumors and was validated through fluorescence and photoacoustic measurements and tissue pathology analysis. The system has the potential to provide real-time, specific detection of intratumor heterogeneity in non-metastatic tumors.

Computer technology has enabled real-time imaging during breast cancer surgery, helping surgeons accurately locate the tumor. However, distinguishing between healthy and cancerous tissue at the resection margin remains a challenge. To address this, a study assessed the use of diffuse reflectance spectroscopy (DRS) for real-time tissue description during breast cancer surgery [60]. An optical biopsy needle with integrated optical fibers was used to obtain DRS data of normal tissue and tumor tissue in 27 patients. The results showed that malignant tissue could be accurately distinguished from healthy tissue, with a Matthews Correlation Coefficient of 0.93 and 0.87, respectively. In a similar vein, S A Kulkarni et al. [61] compared the accuracy of tomosynthesis (DBT), 2D specimen radiography (SR), tomosynthesis (DBT) and full-3D volumetric specimen imager (VSI) in determining the margin status of breast lumpectomy specimens. The pathology results indicated that VSI improved the correlation between the main lumpectomy specimen margin status and surgical pathology, with an area under the curve (AUC) of 0.91 to 0.94, higher than the AUC values of SR and DBT.

### 4.3. Monitoring and assessment of treatment response

The integration of computer technology in monitoring and assessing treatment response in breast cancer has seen significant advancements. The use of medical data mining and analysis techniques, along with the ability to predict the likelihood of recurrence, has enhanced the accuracy and effectiveness of treatment evaluations, achieving better patient outcomes.

Medical data mining and analysis techniques have shown promising accuracy in predicting breast cancer recurrence, with various algorithms including Naive Bayes, RBF Network, J48 and C5.0 being applied. V Chaurasia et al. [62] developed models for predicting breast cancer survivability using data mining algorithms, with Naive Bayes achieving the best performance at 97.36% accuracy, accompanied by RBF Network and J48. In Iran, A Mosayebi et al. [79] utilized data mining techniques to predict breast cancer recurrence, finding that the C5.0 algorithm was the most effective in predicting recurrence in the first to third years, with the most important factors being LN involvement rate, Her2 value, tumor size and free or closed tumor margin. Furthermore, S Simsek et al. [80] presented a hybrid data mining methodology that showed the importance of considering changing variables over time in predicting breast cancer survival, as specific variables change in importance over time and a purely data-driven approach can lead to extremely parsimonious models, providing useful information for medical practitioners to improve cancer care.

**Figure 13.**
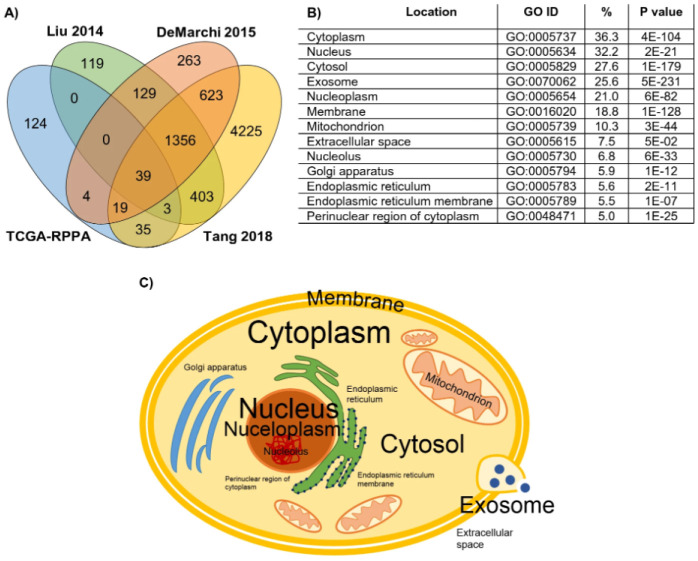
Proteins measured in multiple studies and their cellular localizations. (A) Number of proteins represented in one, two, three or four datasets, (B) proportion of proteins present in various cellular components and (C) graphical representation of cellular origin of the analyzed proteins, where font size is relative to the proportion of proteins from that compartment [63].

Data analysis techniques play a crucial role in investigating the relationship between tumor-infiltrating lymphocytes (TILs) and breast cancer treatment response and predicting patient survival outcomes. C Denkert et al. [81] conducted a study to evaluate the relationship between TILs and neoadjuvant chemotherapy response in primary breast cancer patients. The study demonstrated that higher TIL concentration was correlated with improved response to chemotherapy across all molecular subtypes, but a negative prognostic factor for survival in luminal-HER2-negative subtype. These findings suggest the potential for immune-modulating therapies in breast cancer treatment, but further research is needed to understand the interplay between the immune system and distinct forms of endocrine therapy in luminal breast cancer. W D Lindsay et al. [82] compared the effectiveness of statistical models, such as random forests and logistic regression, in predicting treatment failure and adverse events in breast cancer patients using electronic medical record (EMR) data. Their results indicated that ensemble methods, such as random forests, outperformed single-model methods, such as decision trees and logistic regression, in predicting outcomes, with the patient's medical history being the most significant factor in predicting treatment outcomes. Notably, Á Ösz et al. [63] executed an investigation to compare the expression of four protein biomarkers in breast cancer patients using immunohistochemistry and proteome-level technologies, with the aim of identifying new prognostic biomarkers (Figure 13). The study analyzed data from four disconnected cohorts of 1229 breast cancer patients and found a remarkable association between the levels of biomarkers determined by immunohistochemistry and proteomic methods. Additional candidate proteins were validated as prognostic biomarkers, and a web tool was expanded to integrate the proteomic data examined in this study.

### 4.4. Precision medicine and molecular prognosis

Precision medicine, also known as personalized medicine, is a medical approach that utilizes genetic and molecular information about an individual to inform medical decision-making, including disease diagnosis and treatment. Precision medicine takes into account an individual's unique genetic and molecular profile, as well as their personal and environmental factors, to develop targeted, individualized treatment plans. This approach aims to improve the accuracy and effectiveness of healthcare by reducing guesswork and increasing the chance of success in treating disease.

Precision medicine holds great potential for personalized and effective breast cancer treatment, through the use of mathematical modeling and machine learning (ML) to consolidate biomarker changes and improve risk prediction accuracy. In their work, M T McKenna et al. [83] emphasize the need for a mathematical modeling toolkit in medical oncology to optimize patient treatment selection and schedules, given the current lack of an efficient method to consolidate biomarker changes into a comprehensive understanding of treatment response. They suggest that mathematical modeling can enhance the application of anticancer therapeutics in precision medicine, using breast cancer as an example of how modeling has already shaped current treatment approaches. C Ming et al. [84] evaluate machine learning algorithms against two established breast cancer risk prediction models, BCRAT and BOADICEA, and demonstrate that ML methods outperform these standard models in discriminatory accuracy, with predictive accuracy reaching up to 90% in Swiss clinic-based samples and 88% in US population-based samples. The enhanced accuracy of ML has important implications for personalized medicine, enabling better prevention strategies and individualized clinical management. F Acconcia's [85] recent work explores the *α*-character of computer technology in breast cancer treatment, specifically focusing on the molecular classification of pharmacological treatments, such as anti-estrogen therapy and anti-cancer drugs. The study also assesses the potential of repurposing existing drugs for breast cancer treatment, and examines the Na/K ATPase isoform as a biomarker for ER*α*-positive breast cancer treatment. The study outcomes indicate that cardiac glycosides could be administered in conjunction with other anti-cancer drugs for more effective treatment of ER*α*-positive breast cancer.

Molecular prognosis is a method of predicting cancer progression and survival by analyzing molecular signatures within cancer cells. Molecular prognosis can help doctors better evaluate the severity of cancer, select more appropriate treatment options and predict patient outcomes. Molecular prognosis is generally performed by analyzing genetic mutations, protein expression and signaling pathway activity in tumor samples to assess the biological characteristics and trends of cancer.

Personalized circulating tumor DNA (ctDNA) profiling has emerged as an effective tool for detecting breast cancer recurrence, allowing for high accuracy in residual disease detection and aiding in clinical decision making for breast cancer patients in China. R C Coombes et al. [86] demonstrated the use of ctDNA profiling in detecting breast cancer recurrence, detecting ctDNA in 89% of relapsing patients and finding it absent in all non-relapsing patients. This ctDNA analysis provides a subtle and detailed approach to disease supervision with a lead time of up to 2 years. B R McDonald et al. [87] discussed a new method for detecting residual cancer DNA in plasma, targeted digital sequencing (TARDIS), which overcomes the limitations of current methods in detecting residual disease after treatment in non-metastatic cancer patients. TARDIS demonstrated a high degree of precision in evaluating molecular response and residual disease in the course of neoadjuvant therapy in 80 plasma samples from 33 women diagnosed with stage I to III breast cancer. By improving ctDNA detection by 100 times, personalized ctDNA tracking has become a promising tool to tailor clinical management strategies for patients with curable cancer. A multidimensional model was developed using extreme gradient boosting, which was assessed for its ability to predict disease progression, cancer-specific mortality and all-cause mortality [88]. The model showed high discriminatory power and good calibration, and performed similarly to or better than the PREDICT model in different subgroups, making it a useful tool for predicting prognosis and making clinical decisions for patients with breast cancer in China.

## 5. Discussion

Breast cancer poses a number of diagnostic and treatment challenges, in part because of its variable appearance and the subjectivity involved in interpreting medical images [89]. The accurate identification and diagnosis of breast cancer, particularly in its early stages, can be difficult due to the various forms it can present. The interpretation of diagnostic images, such as mammograms and ultrasounds, can also be influenced by a doctor's experience, training and personal biases, leading to potential inaccuracies in diagnoses. Furthermore, the treatment of breast cancer can be complex, with a range of options available, including surgery, radiation therapy and chemotherapy [90]. A doctor must consider a variety of factors, such as the phase and type of cancer, the patient's age and overall health and the underlying side effects of treatment, when designing a tailored treatment plan [91]. These considerations require a high level of expertise and experience, as well as the ability to effectively communicate with patients and provide them with support and guidance.

Breast cancer diagnosis and treatment have undergone significant changes with the integration of computer-aided technologies [1], [92], [93]. Through a systematic review of the relevant literature, we identified three main areas of interest: machine learning, augmented and virtual reality, and data mining [29], [70], [74], [80]. Machine learning offers improved precision and consistency through the automatic analysis of vast amounts of medical data and images [29], [30]. Advanced algorithms in machine learning can enhance diagnostic accuracy and early detection of breast cancer, thus prolonging patient survival [31]. Augmented and virtual reality provide a more interactive and immersive experience for both patients and medical professionals, and can be utilized for surgical guidance and navigation, education and training, as well as preoperative planning and simulation [70]–[73]. Data mining and analysis can also be utilized to predict breast cancer recurrence and forecast patient survival outcomes, thereby assisting medical professionals in making informed treatment decisions and improving patient outcomes [62], [79]. The integration of these cutting-edge technologies has the potential to revolutionize breast cancer management by offering more personalized and effective care for patients.

However, while the introduction of these technologies holds potential, their wider integration into the healthcare system presents public health challenges. The costs associated with the adoption and maintenance of these technologies can pose significant barriers, especially in low-resource settings [94]. Furthermore, the infrastructure required, from robust computing systems to specialized equipment, might not be readily available everywhere. Additionally, there remains a learning curve for physicians in accepting and adapting to these technologies [9]. The perceived reliability of computer-aided systems, potential malfunctions and the shift from traditional methods could lead to resistance among some healthcare professionals. As we emphasize the potential of these technologies, it's crucial to address these challenges and seek solutions for broader, equitable adoption.

The future of the field of computer-aided diagnosis and treatment in breast cancer management holds much promise. Further advancements in technology and data analysis have the potential to greatly improve the accuracy, efficiency and personalization of breast cancer diagnosis and treatment. As such, the future of research in this field should prioritize the refinement and improvement of existing technologies, as well as exploring new and innovative approaches to diagnosis and treatment. Precision medicine, which takes into account a patient's unique characteristics and medical history, is a promising trend that deserves increased attention [83]–[85]. By leveraging the latest technological advancements, researchers can continue to make strides towards the goal of providing more targeted and effective treatments for patients, leading to improved outcomes and reduced burden of breast cancer. Therefore, the future of breast cancer diagnosis and treatment is poised to be shaped by the continued growth and integration of precision medicine and computer-aided technologies. With a focus on individualized and targeted approaches, there is much reason for optimism in this field.

This systematic review sheds light on the recent advancements in the field of computer-aided diagnosis and treatment in breast cancer management. From a practical standpoint, these advancements have far-reaching implications. By incorporating machine learning and data mining into the diagnostic process, medical practitioners can make well-informed decisions based on a more comprehensive and accurate understanding of the patient's condition. Furthermore, the use of augmented reality and virtual reality can enhance surgical planning and navigation, thus potentially lowering the risk of complications and enhancing patient outcomes. However, it's paramount that these advancements are assessed from a public health perspective, taking into account their cost, accessibility and acceptability among healthcare professionals. To sum up, the recent advancements in computer-aided diagnosis and treatment in breast cancer management have the potential to significantly enhance the accuracy and efficiency of breast cancer diagnosis and treatment, and provide new and innovative ways to tackle this complex and challenging disease.

## 6. Conclusions

In conclusion, the systematic review of recent advancements in the field of computer-aided diagnosis and treatment in breast cancer management underscores the crucial role of technology in healthcare. With the integration of machine learning, augmented reality and virtual reality, and data mining, medical professionals are now able to make more informed decisions and provide more accurate diagnoses. These advancements have the potential to enhance diagnostic accuracy, facilitate earlier detection of breast cancer and streamline treatment processes. In addition, the focus on precision medicine in this field offers a promising trend, as it enables the customization of treatment plans based on a patient's unique characteristics and medical history. This approach has the potential to significantly improve patient outcomes and quality of life. This systematic review underscores the importance of continued investment in technology and research in the field of computer-aided diagnosis and treatment in breast cancer management, which can lead to substantial advancements in the accuracy and efficiency of breast cancer diagnosis and treatment.

## Use of AI tools declaration

The authors declare they have not used Artificial Intelligence (AI) tools in the creation of this article.
